# Case of Emphysema, Brought on by Severe Labour Pains

**Published:** 1792

**Authors:** R. B. Blagden

**Affiliations:** Surgeon at Petworth in Sussex.


V. *Cafe of Emphyfema, brought on by fevere Labour Pains. Communicated in a Letter to Dr.* Simmons *by Mr.* R. B. Blagden, *Surgeon at Petworth in Suffex.*

IN November, 1779, I was a witnefs to a cafe of emphyfema, brought on by fevere labour pains. As nothing of the kind has fince fallen within my obfervation, and as I find from a paper of your own, in the firft volume of the Medical Communications, that thefe cafes are extremely rare, I offer you, with fome little confidence, for the next volume of Medical Facts and Obfervations, an account of it, drawn up at the time when it happened.

The fubject of this cafe was a fmall woman, twenty-five years of age, and this her firft labour. The whole pelvis (and the arch of the os pubis in particular) approached to that of the male, and the external parts were remarkably ſmall and rigid. Notwithſtanding the moſt violent and quickly-repeated pains, the child was not expelled till full ſeven hours after the complete dilatation of the os uteri; its head was wonderfully elongated, and all the means of recovery were ineffectual.

In the moment of its expulſion the woman, with inexpreſſible terror, exclaimed, "I ſhall "be ſuffocated." I haſtened to the other ſide of the bed, when I was ſtruck with her appearance, which was entirely altered, her face, neck, and breaſts, being inflated to an amazing degree.

The emphyſema of the upper and fore part of the neck made, on preſſure, the crackling noiſe which characteriſes this affection; but the emphyſema of the face and the breaſts was perfectly hard and unyielding.

I inſtantly bled her freely, which immediately relieved the ſenſe of ſuffocation, and in ſome meaſure diminiſhed the inflation of the face: ſhe was, nevertheleſs, unable to open her eyes till the fourth day.

The

The whole ſwelling ſubſided very gradually, and was entirely gone in about a week, but the crackling noiſe was plainly felt, for nine or ten days, juſt below the clavicles ; indeed there was air, in ſmall portions, perceivable in the cellular membrane of the arms for many weeks afterwards.

Friction with oil was recommended from the moment of the attack.

The placenta came eaſily away in about half an hour after the blood was drawn : the patient took a gentle purge on the ſecond day after delivery, and another on the fourth ; ſhe had no milk fever, nor any other ſymptom to retard her recovery.

If the bleeding had not given ſuch inſtant relief, the urgency of the ſymptoms would have led me to attempt it, without loſs of time, by inciſion, as practiſed by the late Dr. Hunter (and long before, in an inſtance related by Paré) in a caſe of emphyſema deſcribed in the ſecond volume of Medical Obſervations and Inquiries.

It afterwards appeared that my patient had, for ſome hours, buried her face, during every pain, in the woollen coat of a woman who ſat on the ſide of the bed.

I beg leave to obferve, that I have fince attended the fame perfon in three labours, and that, although they were all of the laborious kind, nothing emphyfematous occurred in either of them.

*Petworth*,

Auguft 30th, 1791.

